# Age and Gender Differences in Facial Attractiveness, but Not Emotion Resemblance, Contribute to Age and Gender Stereotypes

**DOI:** 10.3389/fpsyg.2017.01704

**Published:** 2017-09-29

**Authors:** Rocco Palumbo, Reginald B. Adams, Ursula Hess, Robert E. Kleck, Leslie Zebrowitz

**Affiliations:** ^1^Department of Psychology, Brandeis University, Waltham, MA, United States; ^2^Department of Psychology, University of Chieti, Chieti, Italy; ^3^Department of Psychology, The Pennsylvania State University, State College, PA, United States; ^4^Department of Psychology, Humboldt Universität zu Berlin, Berlin, Germany; ^5^Department of Psychological and Brain Sciences, Dartmouth College, Hanover, NH, United States

**Keywords:** face perception, emotion resemblance, traits impression, connectionist models, aging, stereotypes, facial expression, attractiveness

## Abstract

Considerable research has shown effects of facial appearance on trait impressions and group stereotypes. We extended those findings in two studies that investigated the contribution of resemblance to emotion expressions and attractiveness to younger adults (YA) and older adults (OA) age and gender stereotypes on the dimensions of warmth and competence. Using connectionist modeling of facial metrics of 240 neutral younger and older faces, Study 1 found that, neutral expression older faces or female faces showed greater structural resemblance to happy expressions and less resemblance to angry expressions than did younger or male faces, respectively. In addition, neutral female faces showed greater resemblance to surprise expressions. In Study 2, YA and OA rated the faces of Study 1 for attractiveness and for 4 traits that we aggregated on the dimensions of competence (competent, healthy) and warmth (trustworthy, not shrewd). We found that YA, but not OA, age stereotypes replicated previous research showing higher perceived warmth and lower perceived competence in older adults. In addition, previously documented gender stereotypes were moderated by face age for both YA and OA. The greater attractiveness of younger than older faces and female than male faces influenced age and gender stereotypes, including these deviations from prior research findings using category labels rather than faces. On the other hand, face age and face sex differences in emotion resemblance did not influence age or gender stereotypes, contrary to prediction. Our results provide a caveat to conclusions about age and gender stereotypes derived from responses to category labels, and they reveal the importance of assessing stereotypes with a methodology that is sensitive to influences of group differences in appearance that can exacerbate or mitigate stereotypes in more ecologically valid contexts. Although the gender differences in attractiveness in the present study may not have generalizability, the age differences likely do, and the fact that they can weaken the attribution of greater warmth and strengthen the attribution of lower competence to older than younger individuals has important practical implications.

## Introduction

Cultural wisdom instructs us not to judge a book by its cover. This warning suggests both that our natural inclination is to judge people by their appearance and also that doing so will lead to adverse effects. Both are true. On the first point, trait impressions from faces are fast and automatic, elicited by exposure as brief as 100 ms or less (Willis and Todorov, 2006). Moreover, there is remarkable consensus in trait impressions from faces that extends across diverse cultures (Zebrowitz et al., 2012) and shows similarities across the lifespan (Keating and Bai, 1986; Montepare and Zebrowitz-McArthur, 1989; Langlois et al., 1990; Zebrowitz et al., 2013; Cogsdill et al., 2014). The second point implied by the caution against judging people by their appearance is supported by evidence that this does yield adverse effects by contributing to race, gender, and age stereotypes. The fundamental dimensions underlying trait impressions are warmth and competence (Rosenberg et al., 1968), which capture both trait impressions from people's faces (Todorov et al., 2015) and group stereotypes (Cuddy et al., 2008). The present research investigated the contribution of variations in facial appearance to age and gender stereotypes on these dimensions, which has not been previously addressed.

### Age stereotypes and contributions of appearance

Age stereotypes are manifested in the attribution of similar traits to older vs. younger people in the absence of meaningful individuating information. There is considerable evidence for negative stereotypes of older people across many cultures (Nelson, 2002; Löckenhoff et al., 2009; North and Fiske, 2015). These include negative stereotypes regarding the competence of older adults, such as the perception that aging is associated with declines in competence at performing everyday tasks and new learning (Löckenhoff et al., 2009). Other research has conceptualized age stereotypes on the dimensions of warmth and competence (Cuddy and Fiske, 2002; Cuddy et al., 2005). This work found that trait impressions of older adults based on their category membership were more negative than impressions of younger adults on the dimension of competence, including the traits “skillful” and “able,” but more positive on the dimension of warmth, including the traits “trustworthy” and “sincere.” These results led to the conclusion that elderly people are stereotyped as “doddering, but dear” (Cuddy and Fiske, 2002). However, the question remains as to whether these age stereotypes generalize to those elicited by actual people rather than category labels. The present study begins to fill this gap in the literature.

A previous study that assessed impressions of photographs of neutral expression older and younger faces, rather than using category labels, found that older faces were judged less energetic and less growth-oriented (Ebner, 2008). Other reseearch using photographs has found positive as well as negative impressions of older people. Specifically, some photographs of older people evoked positive stereotypes while others evoked negative stereotypes (Brewer et al., 1981). However, the physical attributes that elicited the varying impressions of older adults were not identified in this work, and the stereotypes themselves were not explictly mapped onto the well-established competence and warmth dimensions of trait impressions. The present study addressed these issues.

Previous research examining stereotypes of the elderly also found that older adults were judged to be less attractive (Ebner, 2008; Löckenhoff et al., 2009), and other research has shown that age stereotypes are linked not simply to chronological age, but also to physical appearance. Specifically, unattractive physical qualities, such as wrinkling, gray hair, and baldness, are associated with more negative impressions of elderly faces (Hummert, 1994; Muscarella and Cunningham, 1996; Hummert et al., 1997). In addition, Zebrowitz et al. (2003) found that, compared with younger faces, older faces showed greater resemblance to faces with genetic anomalies and this contributed not only to impressions of older faces as less attractive, but also to impressions of them as less healthy, sociable, and intelligent than younger faces. More generally, the well documented attractiveness halo effect (Eagly et al., 1991) provides reason to believe that the lower attractiveness of older faces would augment negative stereotoypes, like incompetence, and weaken positive stereotypes, like warmth. Older and younger faces differ in many ways besides attractiveness. One that will be examined in the present research is a possible difference in their resemblance to emotion expressions. Research has documented an influence of emotion resemblance on impressions of warmth and competence (Zebrowitz et al., 2007, 2010) and, as discussed more fully below, there is reason to expect differences between younger and older faces.

### Gender stereotypes and contributions of appearance

The dimensions of warmth and competence capture gender stereotypes as well as age stereotypes, with men perceived as higher than women in competence, and women perceived as higher in warmth (Eagly and Steffen, 1984; Fiske et al., 2002; Cuddy et al., 2008). Although this work has examined stereotypes based on gender labels, other research has demonstrated that male-female differences in appearance contribute to the stereotypes. Indeed, Deaux and Lewis (1984) found that physical appearance was the single most influential component of sex-role stereotypes. Participants inferred traits that were consistent with a description of the target's body build even when those inferences were inconsistent with those associated with the target's gender label. Another study showed that sex stereotypes are also influenced by typical sex differences in facial appearance. Women's faces are more neotenous than men's (Enlow, 1990), and these variations in babyfaceness have a strong effect of gender stereotypes. When facial maturity was typical (babyfaced women, maturefaced men), the women were perceived as warmer and less competent than the men. However, when the male faces were manipulated to be babyfaced and the female faces to be maturefaced, the gender stereotyped attribution of warmth was eliminated and the women were perceived as more competent than the men (Friedman and Zebrowitz, 1992). As discussed in more detail below, babyfaceness is related to emotion resemblance (Marsh et al., 2005; Zebrowitz et al., 2007), and research investigating differences in the emotion resemblance of neutral expression male and female faces has found that female faces are more similar than male faces to happy and surprised expressions and less similar to angry expressions (e.g., Becker et al., 2007; Zebrowitz et al., 2010).

The aim of the work to be reported here was to investigate how variations in facial appearance moderate age and gender stereotypes. In Study 1, using connectionist modeling, we assessed differences in the resemblance to emotion expressions of neutral expression younger and older male and female faces. We sought to determine whether an objective measure of emotion resemblance, free from cultural expectations, is related to age differences. In addition, we sought to replicate previous evidence for objective differences in the emotion resemblance of male vs. female faces and extend that evidence to older faces. In Study 2, we examined the contribution of face age and sex differences in emotion resemblance to YA and OA age and gender stereotypes. Furthemore, considering that previous studies found that lower facial attractiveness is associated with more negative impressions and that older adults tend to be judged less attractive, we also investigated the contribution of group differences in attractiveness to these age related stereotypes.

## Study 1: face age and sex differences in resemblance to emotion expressions

Study 1 investigated face age and sex differences in resemblance to happy, angry, and surprised expressions. We examined these expressions because, as discussed in more detail in the introduction to Study 2, each has previously been shown to influence impressions of warmth and competence, which are the two strongest components of age and gender stereotypes.

Previous research has documented similarities between facial expressions of emotion and neutral expression faces from various demographic groups. Faces of babies resemble surprise and fear expressions more than do faces of adults, and babies show less resemblance to anger, effects that have been demonstrated both by subjective ratings of the faces (Marsh et al., 2005) and by connectionist modeling using facial metrics (Zebrowitz et al., 2007). Consistent with evidence that women's faces are more neotenous than men's (Enlow, 1990), female faces are associated with surprise expressions and male faces with angry ones (Le Gal and Bruce, 2002; Becker et al., 2007; Zebrowitz et al., 2010). There is also some evidence that female neutral faces resemble happy expressions more than do male faces (Hess et al., 1997; Becker et al., 2007).

Whereas gender comparisons and age comparisons involving infants vs. adults have shown that emotion resemblance varies across these demographic groups, comparisons of older and younger adult faces are less clear. Research using subjective assessments of resemblance have found that neutral expression older faces are likely to be misperceived by younger raters as any one of several emotion expressions (Malatesta et al., 1987; Ebner, 2008; Freudenberg et al., 2015). Similarly, automated emotion recognition software (CERT) that registers the intensity of different facial action units yielded lower probability estimates that neutral expressions were neutral when posed in older than younger faces (Freudenberg et al., 2015). This research also found that older faces with neutral expressions were rated higher in anger, contempt, disgust, and happiness than younger faces, but lower in sadness, with no differences in perceptions of fear or surprise (Freudenberg et al., 2015).

The advantage of an objective measure, like CERT, is that it removes the influence of similarities between the *cultural meaning* of the demographic categories and the emotion expressions, such as the assumption that men are more likely to be angry than women (Hess et al., 1997), thus clearly identifying *structural similarities* between faces from certain demographic categories and those with certain emotion expressions. In the present study, we used connectionist modeling, which is also impervious to stereotyped assumptions about the emotion resemblance of different demographic groups. Whereas CERT registers facial action units associated with emotion expressions, connectionist modeling assesses whether facial metrics, such as eye height, nose width, and chin length reveal greater structural similarities between older than younger faces and certain emotion expressions. Such structural similarities could be occasioned by age-related bone resorption and/or it could be a by-product of textural changes. For example, sagging upper eyelids may make the eyes look smaller.

We derived our predictions for age differences in emotion resemblance from previous research that used connectionist modeling to investigate the resemblance of elderly and young adult faces to babies, finding elderly faces more similar to babies than young adult faces (Zebrowitz et al., 2003). Although it may be surprising to find that older adults are more baby-faced, age-associated bone loss causes elderly people to have small jaws, double chins, and jowls, just as babies do. Indeed, the characterization of elderly stereotypes as “doddering but dear” (Cuddy and Fiske, 2002) captures the incompetence and warmth that characterizes impressions of babies. Given that elderly faces resemble babies more than do young adult faces, and that baby faces resemble anger less than young adult faces (Marsh et al., 2005; Zebrowitz et al., 2007), we predicted that elderly faces would also resemble anger expressions less than young adult faces. Although our previous research found that faces of babies and young adults did not differ in resemblance to happy faces, this may have been due to the large eyes that characterize babies, in contrast to the squinting eyes of a smile that may be more characteristic of elderly adult eyes. This, coupled with evidence from CERT that older faces resemble happy expressions more, led us to predict that older faces would resemble happy expressions more than younger ones. In addition, we thought that, unlike babies with wide eyes, older faces may not resemble surprise faces more, even though babies, as compared with adults, do. Finally, we expected to replicate previous findings that female faces resemble happy and surprise expressions more than male faces do, with the reverse for angry faces, and we expected these results to be extended to older as well as younger faces.

### Method

Connectionist models were trained to recognize the facial metrics of anger, happy, and surprise expressions in Caucasian male and female young adult training/test faces. The extent to which the models detected similarities to these emotions in neutral expression faces was then examined using a separate set of younger and older generalization faces.

#### Faces

##### Training/test faces

Training/test faces were taken from a previous study (Zebrowitz et al., 2007). They included digitized black and white portrait photos of 26 Caucasian men and 26 Caucasian women in their 20s or 30s, each of whom posed neutral, happy, angry, and surprise expressions. Previously reported validations of the database demonstrated significantly higher ratings of anger for angry faces than each of the other categories, higher ratings of happy for happy faces than each of the other categories, and higher ratings of surprise for surprise faces than each of the other categories (Zebrowitz et al., 2007).

##### Generalization faces

Generalization faces included 120 older and 120 younger neutral expression faces, with men and women equally represented within each age group. All faces were Caucasian. The entire set of 240 faces was selected from three different databases: 105 neutral expression faces (47 older faces) were selected from the FACES database (Ebner et al., 2010) which comprises digital high quality, front-view photographs of three different age groups; 8 neutral expression older faces were selected from the Humboldt face set (Fölster et al., 2015). The remaining 127 neutral expression faces (65 older faces) were selected from The Center for Vital Longevity Face Database (Minear and Park, 2004) created at the University of Michigan. The younger faces were photographed between 18 and 31 years of age (*M* = 23.06, *SD* = 3.22) and the older faces were photographed between 65 and 91 years of age (*M* = 73.42, *SD* = 5.41). We used four criteria for image selection: neutral expression, no head tilt, no eyeglasses, and no beards. To verify that faces had neutral expressions, four judges (2 males) provided smile ratings on a 5-point scale with endpoints labeled 1 = no smile and 5 = big smile. All faces were shown in gray-scale.

**Figure 1:**
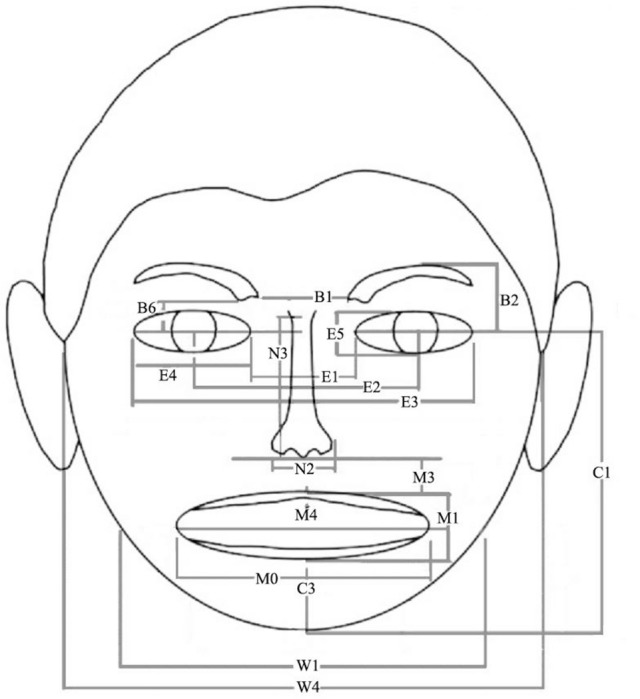
Location of facial metrics used as inputs to the connectionist models trained on facial metrics. All metrics were normed by E2, interpupil distance. B1, Eyebrow separation; B2, Eyebrow height; B6, Distance from lower inner corner eyebrow and top of eye; E1, Eye separation; E2, Interpupil distance (used to normalize other measures); E3, Distance between outer corners of eyes; E4, Horizontal eye width; E5, Eye height; C1, Chin to pupil height; C3, Chin length; M0, Mouth width; M1, Lip thickness; M3, Distance from end of nose to middle top of upper lip; M4, Upper lip thickness; N2, Nose width; N3, Nose length; W1, Jaw width; W4, Face width.

Following the procedure reported by Zebrowitz et al. (2007), in house software was used to mark 64 points on digitized images of each face from which facial metrics were computed using automatic procedures written in Visual Basic and Excel (Figure 1). After establishing reliability (>0.7) for points marked by two judges on a random subset of 24 faces for each category, one judge marked the remaining faces and those points were used to calculate the final facial metrics. Eighteen non-redundant facial metrics were selected as full facial inputs to the connectionist model. These included facial roundness plus the metrics shown in Figure 1. Facial roundness was computed by determining the average of the radii of two circles—one created by connecting facial points 31 right, 35 right, and 12, and another circle connecting facial points 31 left, 35 left and 12, with a smaller average radius signifying more roundness. To adjust for variations in distance from the camera, each facial metric was normalized by an additional metric, inter-pupil distance (E2) (Zebrowitz et al., 2007).

#### Connectionist modeling

The *total set* of faces used to train the network was composed of 208 faces (52 each for neutral, surprise, angry, happy expressions). Networks were trained to differentiate either happy from neutral faces, surprise from neutral faces, or angry from neutral faces. On each of 20 trials, 34 neutral and 34 emotion faces (either happy, surprise, or angry faces) were randomly selected from the total set to compose the *training set*, with a different random set of faces selected on each trial. The remaining 18 faces in each of the two categories composed the *test set*. The modeling had three phases. First, in the *training phase*, the 18 reliable facial metrics were provided as input to artificial neural networks that were trained with supervised learning to differentiate the 68 training faces (34 men), half with a neutral expression and half with an emotion expression. In the second or *test phase*, the trained network was tested on the set of 36 test faces (18 men) that differed in the emotion on which the network had been trained in order to establish that training was successful. In the third, *generalization phase*, the trained network was provided with input metrics from the 240 neutral expression generalization faces, and the extent to which the output units responded to each of these faces was determined. These three phases were repeated for 20 trials to establish a reliable index of network activation by each face. Performing the entire procedure for networks trained to differentiate neutral expression faces from each of the three different emotion expressions generated three dependent variables for each generalization face: average activation across 20 trials of the happy output unit, the surprise output unit, and the angry output unit.

The connectionist models were standard back-propagation neural networks with one input layer, one hidden layer, and one output layer. Each input node projected to any or all of the hidden nodes and the hidden nodes projected to the two output units (neutral and one of the emotions). The input weight matrices connecting the layers consisted of numbers between –1 and 1. The output units were rescaled into graded values ranging from 0 to 100% activation. All units were nonlinear and mapped the weighted sum of their inputs to their output using a sigmoidal transfer function. The training parameters were 4 hidden nodes, 3,000 training epochs per trial, a 0.02 learning rate, and a 0.2 error goal.

### Results

#### Reliability of facial metrics

High inter-judge agreement for the facial metrics of the emotion expression faces (cf. training and test set) was previously reported by Zebrowitz et al. (2007). The selected input metrics for the faces in the generalization set also showed high agreement both for the normalization interpapillary distance, *r* = 0.92, and the selected input metrics (>0.786; mean *r* = 0.87).

#### Network training

##### Surprise-neutral networks

Training a network to differentiate surprise and neutral faces achieved 92.43% correct identification of the 68 training faces and 85.14% correct identification of the 36 test faces, averaged across 20 trials, with significantly higher activation of the surprise unit by surprise faces (*M* = 82.82, *SD* = 17.30) than neutral ones (*M* = 18.18, *SD* = 12.38), *F*_(1, 102)_ = 479.79, *p* < 0.001, η^2^ = 0.825.

##### Anger-neutral networks

Training a network to differentiate angry and neutral faces achieved 88.31% correct identification of the 68 training faces and 72.78% correct identification of the 36 test faces, averaged across 20 trials with activation of the angry unit significantly higher for angry faces (*M* = 73.97, *SD* = 24.69) than for neutral ones (*M* = 22.55, *SD* = 16.63), *F*_(1, 102)_ = 155.07, *p* < 0.001, η^2^ = 0.603. It should be noted that the less successful training of the anger- than the happy- or surprise-neural networks is consistent with human judges' ratings of the faces. Zebrowitz et al. (2007) found that the neutral faces were rated higher in anger than in happiness or surprise. Also, although neutral faces were rated the lowest of all expressions in surprise and happiness, they were rated second only to anger faces in anger, a finding that is consistent with other evidence concerning similar reactions to neutral and anger expressions (e.g., Vrana and Gross, 2004).

##### Happy-neutral networks

Training a network to differentiate happy and neutral faces achieved 92.75% correct identification of the 68 training faces and 86.39% correct identification of the 36 test faces, averaged across 20 trials with activation of the happy unit significantly higher for happy faces (*M* = 79.89, *SD* = 16.60) than for neutral ones (*M* = 19.14, *SD* = 11.87), *F*_(1, 102)_ = 460.10, *p* < 0.001, η^2^ = 0.819.

#### Smile ratings of generalization faces

A 2 (Face Age) × 2 (Face Sex) ANOVA on the smile ratings for these faces revealed no significant effect of face age (*M* = 1.37, *SD* = 0.49 and *M* = 1.28, *SD* = 0.41, for older adults and younger adults respectively), *F*_(1, 236)_ = 2.36, *p* = 0.125, η^2^ = 0.010, no significant effect of face sex (*M* = 1.30, *SD* = 0.48 and *M* = 1.33, *SD* = 0.42, for men and women respectively), *F*_(1, 236)_ = 0.22, *p* = 0.642, η^2^ = 0.001, and no significant face age × face sex interaction, *F*_(1, 236)_ = 0.56, *p* = 0.453, η^2^ = 0.002. The final set of 240 faces (see footnote^1^) consisted of 120 Older faces (60 male) and 120 Younger faces (60 male).

#### Effects of face age and sex on emotion resemblance

The means and *SD*s in Table 1 shows how much neutral expression faces of each age and sex activated the network units trained to recognize angry, happy, and surprise faces. A higher activation of the network unit signifies higher resemblance of the face to the emotion for which the network is trained.

**Table 1:** Effects of face age and sex on emotion resemblance.

**Face**	**Surprise**		**Angry**		**Happy**	
	**Mean**	***SD***	**Mean**	***SD***	**Mean**	***SD***
Old	23.58	13.24	43.96	13.96	34.38	12.14
Young	21.53	13.31	53.99	16.88	17.26	8.47
*F*_(1, 236)_	1.444		26.332^****^		166.424^****^	
Female	24.35	14.78	45.43	16.07	27.99	13.69
Male	20.76	11.38	52.53	15.71	23.65	13.04
*F*_(1, 236)_	4.445^*^		13.190^****^		10.649^***^	

*N = 240*,

* *p < 0.05*,

*** *p < 0.005*,

**** *p < 0.001*.

##### Surprise unit activation

Face Age had a no significant effect on activation of the network unit trained to recognize surprise faces, *F*_(1, 236)_ = 1.44, *p* = 0.231, η^2^ = 0.006 (*M* = 23.58, *SD* = 13.24 and *M* = 21.53, *SD* = 13.31 for older and younger faces, respectively), while female faces activated this network unit (*M* = 24.36, *SD* = 14.78) significantly more than did male faces (*M* = 20.76, *SD* = 11.38), *F*_(1, 236)_ = 4.44, *p* = 0.036, η^2^ = 0.018. There was no significant face age × face sex interaction, *F*_(1, 236)_ = 0.22, *p* = 0.636, η^2^ = 0.001. These results indicate that neutral expression female faces resemble surprise expressions more than do male faces.

##### Anger unit activation

Neutral expression younger faces activated the network unit trained to recognize angry faces (*M* = 53.99, *SD* = 16.88) significantly more than did older faces (*M* = 43.96, *SD* = 13.96), *F*_(1, 236)_ = 26.33, *p* < 0.001, η^2^ = 0.100, and male faces activated this network unit (*M* = 52.53, *SD* = 15.71) significantly more than did female faces (*M* = 45.43, S*D* = 16.07), *F*_(1, 236)_ = 13.19, *p* < 0.001, η^2^ = 0.053. There was no significant face age × face sex interaction, *F*_(1, 236)_ = 0.12, *p* = 0.731, η^2^ = 0.001. These results indicate that neutral expression younger faces and male faces resemble anger more than do older faces and female faces, respectively.

##### Happy unit activation

Neutral expression older faces activated the network unit trained to recognize happy faces (*M* = 34.39, *SD* = 12.14) significantly more than did younger faces (*M* = 17.26, *SD* = 8.47), *F*_(1, 236)_ = 166.42, *p* < 0.001, η^2^ = 0.414, and female faces activated this network unit (*M* = 27.99, *SD* = 13.69) significantly more than did male faces (*M* = 23.65, *SD* = 13.04), *F*_(1, 236)_ = 10.65, *p* = 0.001, η^2^ = 0.043. There was no significant face age × face sex interaction, *F*_(1, 236)_ = 0.57, *p* = 0.812, η^2^ < 0.001. These results indicate that neutral expression older faces and female faces resemble happy expressions more than do younger and male faces, respectively.

### Discussion

Study 1 provides objective evidence for differences in the resemblance of older vs. younger and female vs. male neutral expression faces to particular emotions that are not vulnerable to biases introduced by age or gender stereotypes. Specifically, as predicted, the facial metrics of younger faces resemble anger expressions more than do those of older faces and the metrics of older faces resemble happy expressions more than do those of younger faces. Also as predicted, the facial metrics of male faces resemble anger expressions more and happy expressions less than do those of female faces, and male faces resemble surprise expressions less than do female faces.

The effects of face age on anger resemblance are consistent with previous evidence that older faces resemble babies more, since baby faces also show less resemblance to anger than do young adult faces (Zebrowitz et al., 2003). The effects of age on happy resemblance are consistent with recent evidence using a different method of assessing objective resemblance to emotion expressions, CERT (Freudenberg et al., 2015). Although baby faces show more resemblance to surprise than young adult faces, we did not find this effect for older faces, perhaps due to the effects of aging to reduce visible eye size in older adults. The effects of face sex on anger and happy resemblance are consistent with previous research (Hess et al., 1997; Becker et al., 2007; Zebrowitz et al., 2010). In addition, we found that these effects of face sex held true for both older and younger faces, and that the effects of face age held true for both male and female faces, questions that had not been addressed in previous research.

## Study 2: the contribution of emotion resemblance and attractiveness to age and gender stereotypes

In the second study, we used the network's estimate of the probability that each face is showing a particular emotional expression, to investigate the contribution of face age and sex differences in emotion resemblance to age and gender stereotypes, respectively. As noted earlier, emotion resemblance not only varies across demographic categories, but also it contributes to trait impressions and group stereotypes. Specifically, the adaptive value of responding appropriately to emotional expressions, such as avoiding an angry person or approaching a happy one, is overgeneralized to individuals whose facial structure merely resembles a particular emotional expression, with effects on trait impressions of those individuals that extend to group stereotypes (Zebrowitz and Collins, 1997; Zebrowitz et al., 2010; Zebrowitz and Montepare, 2014).

Neutral expression faces that show more resemblance to an angry expression, either as assessed by human raters (Montepare and Dobish, 2003) or by objective methods (Zebrowitz et al., 2007, 2010; Said et al., 2009) are perceived as lower on a warmth dimension and higher on a competence dimension, with opposite impressions of neutral faces showing greater resemblance to a happy expression. Neutral expression faces that show more objective resemblance to a surprise expression also are perceived as less competent and more warm than those with less resemblance to surprise (Zebrowitz et al., 2007). These effects have been documented for both YA and OA judges (Franklin and Zebrowitz, 2013), although resemblance has been assessed only for young adult faces. Pertinent to the current focus on effects of emotion resemblance on age and gender stereotypes, research also has shown that race differences in emotion resemblance contribute to race stereotypes (Zebrowitz et al., 2010).

As noted above, attractiveness also makes a strong contribution to impressions of warmth and competence (Eagly et al., 1991, and this has been documented for OA as well as YA (Zebrowitz et al., 2014). In Study 2 we investigated YA and OA age and gender stereotypes. We also predicted that face age and face sex differences in emotion resemblance and attractiveness would contribute to the stereotypes.

### Age and gender stereotype predictions

Consistent with the literature discussed earlier, we predicted that older faces and female faces would be judged more positively on a warmth dimension but more negatively on a competence dimension, as compared with younger and male faces, respectively. Research investigating whether these stereotypes vary with rater age have yielded mixed results. Some studies examining rater age differences failed to find differences in age stereotypes (e.g., Bailey, 1991; Erber and Rothberg, 1991), while others suggest that older adults (OA) have more positive attitudes toward aging and older faces than do younger adults (YA) (for reviews see Kite et al., 2005; Ebner, 2008). These results led us to predict that any differences between younger and older raters would show more positive responses to older faces by the latter group. We did not predict any age differences in gender stereotypes, since research indicates similar effects across age (Nesbitt and Penn, 2000; Ebert et al., 2014; Siyanova-Chanturia et al., 2015; Strobach and Woszidio, 2015), although none of this work examined impressions from faces.

### Emotion resemblance predictions

The age differences in emotion resemblance documented in Study 1 together with evidence that both happy and surprise resemblance increase perceived warmth, while anger resemblance decreases it (Zebrowitz et al., 2007, 2010) and that both anger and surprise resemblance decrease perceived competence (Zebrowitz et al., 2007, 2010) yielded the following predictions: (1) controlling the greater resemblance of older faces and female faces to happy expressions and their lesser resemblance to anger would weaken the perception of higher warmth in older and female faces compared with younger and male faces, respectively; (2) controlling older and female faces lesser resemblance to anger would weaken the perception of lower competence in older and female faces compared with younger and male faces, respectively; and (3) controlling female faces greater resemblance to surprise would weaken the perception of their greater warmth and the perception of their lower competence as compared with male faces.

### Attractiveness predictions

Previous evidence that older faces are less attractive (Zebrowitz et al., 2003; Ebner, 2008; Löckenhoff et al., 2009) led us to expect that they also would be perceived as less attractive than younger ones in our study. We further predicted that: (1) perceptions of greater warmth in older than younger adults would be strengthened when controlling the negative contribution of older adults lesser attractiveness to perceived warmth and (2) perceptions of lesser competence in older than younger adults would be weakened when controlling the negative contribution of older adults' lesser attractiveness to perceived competence. Finally, although we had no reason to expect the male and female faces to differ in attractiveness, any differences would yield similar expectations regarding the effects of controlling attractiveness on impressions of warmth and competence.

### Method

#### Participants

Five groups of 20 OA and 20 YA, with equal numbers of men and women in each group, participated in the study for a total of 100 OA and 100 YA individuals. YA were students recruited from University of Chieti, Italy while OA were recruited from the local community. The study was approved by the local departmental ethical committee. All participants were volunteers and provided their written informed consent.

Each group was asked to rate the 240 faces used in Study 1 on just one of five dimensions: competence; health; naivete; trustworthiness and attractiveness. Although the average age of the OA and YA participants differed slightly across rater groups no age differences between groups were found for either OA raters, *F*_(4, 95)_ = 0.97, *p* = 0.426, η^2^ = 0.039, or YA raters, *F*_(4, 95)_ = 1.70, *p* = 0.157, η^2^ = 0.067.

#### Procedure

Each of the 240 faces used in Study 1 was rated on a 7-point scale for competence (1—not at all competent/per nulla competente; 7—very competent/molto competente); health (1—not at all healthy/ per nulla sano; 7—very healthy/molto sano); shrewedness (1—very naïve/molto ingenuo; 7—very shrewd/molto furbo); trustworthiness (1—not at all trustworthy/per nulla affidabile; 7—very trustworthy/molto affidabile) and attractiveness (1—not at all attractive/per nulla attraente; 7—very attractive/molto attraente). The rating task was administered using OpenSesame version 0.27.3, a graphical open-source experiment builder for the social sciences. Each face was randomly presented for 2 s after which the rating scale appeared. Once participants made their rating, a new face was shown. The experiment lasted approximately 15 min. Inter-Rater Reliability for YA and OA across the 5 ratings are shown in Table 2.

**Table 2:** Inter-rater reliability.

		**α**	**ICC_(2, k)_**	**95% CI**		***F***	***df*1**	***df*2**	***p***
				**Lower**	**Upper**				
YA	Competence	0.686	0.654	0.587	0.714	3.182	239	4541	<0.001
	Health	0.935	0.926	0.911	0.940	15.311	239	4541	<0.001
	Shrewdness	0.745	0.724	0.671	0.773	3.919	239	4541	<0.001
	Trustworthiness	0.824	0.790	0.745	0.830	5.687	239	4541	<0.001
	Attractiveness	0.935	0.903	0.873	0.926	15.396	239	4541	<0.001
	Warmth composite	0.827	0.802	0.761	0.838	5.793	239	4541	<0.001
	Competence composite	0.921	0.912	0.895	0.928	12.658	239	4541	<0.001
OA	Competence	0.906	0.892	0.869	0.912	10.628	239	4541	<0.001
	Health	0.917	0.890	0.861	0.913	12.083	239	4541	<0.001
	Shrewdness	0.635	0.611	0.537	0.678	2.742	239	4541	<0.001
	Trustworthiness	0.830	0.798	0.755	0.836	5.891	239	4541	<0.001
	Attractiveness	0.896	0.853	0.812	0.886	9.604	239	4541	<0.001
	Warmth composite	0.842	0.829	0.795	0.859	6.343	239	4541	<0.001
	Competence composite	0.953	0.912	0.895	0.928	12.658	239	4541	<0.001

*Two-way random effects model. Intraclass correlation coefficients using an absolute agreement definition. ICC average measures*.

### Results

#### Factor analyses of trait impressions

We performed separate factor analyses on OA and YA ratings of the faces, excluding attractiveness, to confirm the 2-dimensional “competence” and “warmth” dimensions documented in previous research. The results for YA were as expected, with competence and health loading on one factor and trustworthy and shrewd (opposite loading) on the second factor. The results for OA deviated from past research that has focused largely on factor structures for YA. Competence, health, and trustworthy ratings all loaded highest on one factor, with shrewd ratings loading on the second factor (Table 3). Since trustworthy ratings loaded on the second factor more strongly than did competence or health ratings, we created the same trait composites for YA and OA to facilitate comparisons across rater age. The “warmth” composite was computed by summing ratings of trustworthy and reverse scores ratings of shrewd; and the “competence” composite was computed by summing ratings of competence and health. Inter-Rater Reliability for YA and OA for the 2 composite scores are shown in Table 2.

**Table 3:** Factor Analyses of trait impressions.

	**YA**		**OA**	
	**Factor 1**	**Factor 2**	**Factor 1**	**Factor 2**
Competence	0.935	−0.103	0.941	−0.230
Health	0.840	0.173	0.937	−0.153
Shrewdness	0.404	0.815	−0.226	0.967
Trustworthiness	0.524	−0.769	0.821	−0.464

*Extraction Method: Principal Component Analysis. Rotation Method: Varimax with Kaiser Normalization*.

#### ANOVAs on trait composites and attractiveness

To identify age and gender stereotypes and their moderation by rater age, we performed rater age × face age × face sex ANOVAs on the competence and warmth composites, with rater age a within face variable. We performed the same analysis on attractiveness ratings to ascertain whether our predictions regarding effects of attractiveness on age stereotypes were warranted. To enhance readability, all main effect and interaction means are shown in Table 4 rather than in the text. Interactions are also depicted in figures.

**Table 4:** Older and younger adults' rating scores Face Age × Face Sex × Rater Age.

		**Face age (FA)**		**Face sex (FS)**		**Rater age (RA)**		FA ^*^ FS				FA ^*^ RA				FS ^*^ RA				FA ^*^ FS ^*^ RA							
																				**YF**				**OF**			
								**YF**		**OF**		**YF**		**OF**		**F**		**M**		**F**		**M**		**F**		**M**	
		**YF**	**OF**	**F**	**M**	**YA**	**OA**	**F**	**M**	**F**	**M**	**YA**	**OA**	**YA**	**OA**	**YA**	**OA**	**YA**	**OA**	**YA**	**OA**	**YA**	**OA**	**YA**	**OA**	**YA**	**OA**
Warmth	M	8.51	8.02	8.52	8.00	8.19	8.33	9.04	7.98	8.01	8.03	8.07	8.95	8.32	7.72	8.42	8.62	7.96	8.05	8.54	9.53	7.60	8.36	8.31	7.70	8.33	7.73
	SD	1.12	1.07	1.11	1.08	1.03	1.21	0.94	1.03	1.03	1.12	0.88	1.13	1.52	0.90	0.91	1.28	1.09	1.06	0.71	0.89	0.78	1.11	1.07	0.89	1.24	0.92
Competence	M	10.09	7.33	8.65	8.77	8.55	8.87	10.24	9.94	7.07	7.60	9.69	10.49	7.41	7.26	8.48	8.83	8.63	8.91	9.75	10.72	9.63	10.25	7.20	6.93	7.63	7.58
	SD	1.08	0.89	1.86	1.52	1.46	1.89	0.98	1.15	0.95	0.75	0.93	1.07	0.89	0.90	1.56	2.10	1.35	1.67	0.80	0.90	1.04	1.17	0.98	0.89	0.73	0.78
Attractiveness	M	4.19	2.88	3.63	3.43	2.90	4.17	4.41	3.96	2.86	2.91	3.53	4.84	2.27	3.50	3.03	4.24	2.77	4.10	3.79	5.03	3.27	4.65	2.26	3.45	2.27	3.55
	SD	1.08	0.76	1.17	1.10	0.97	0.91	0.98	1.13	0.76	0.77	0.95	0.74	0.46	0.44	0.90	0.94	0.87	0.87	0.90	0.57	0.93	0.85	0.51	0.43	0.42	0.44

##### Warmth composite

**Figure 2:**
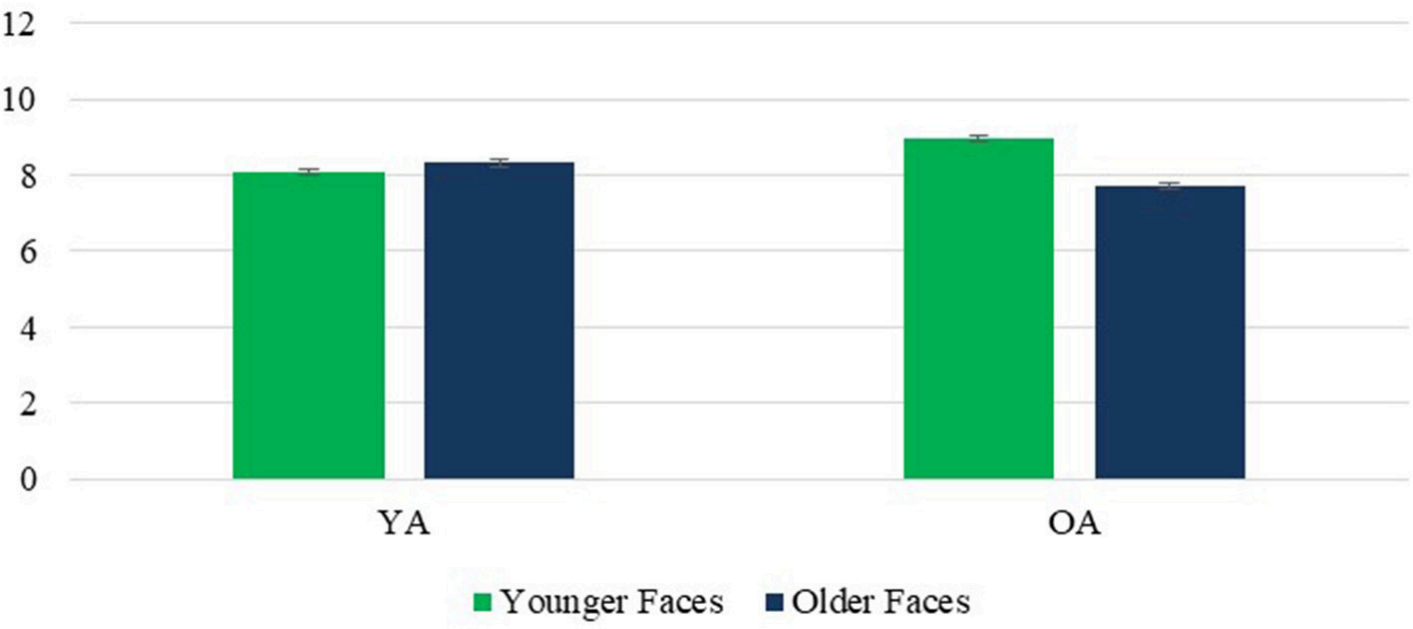
Warmth: Face Age × Rater Age interaction. Error bars are SE.

**Figure 3:**
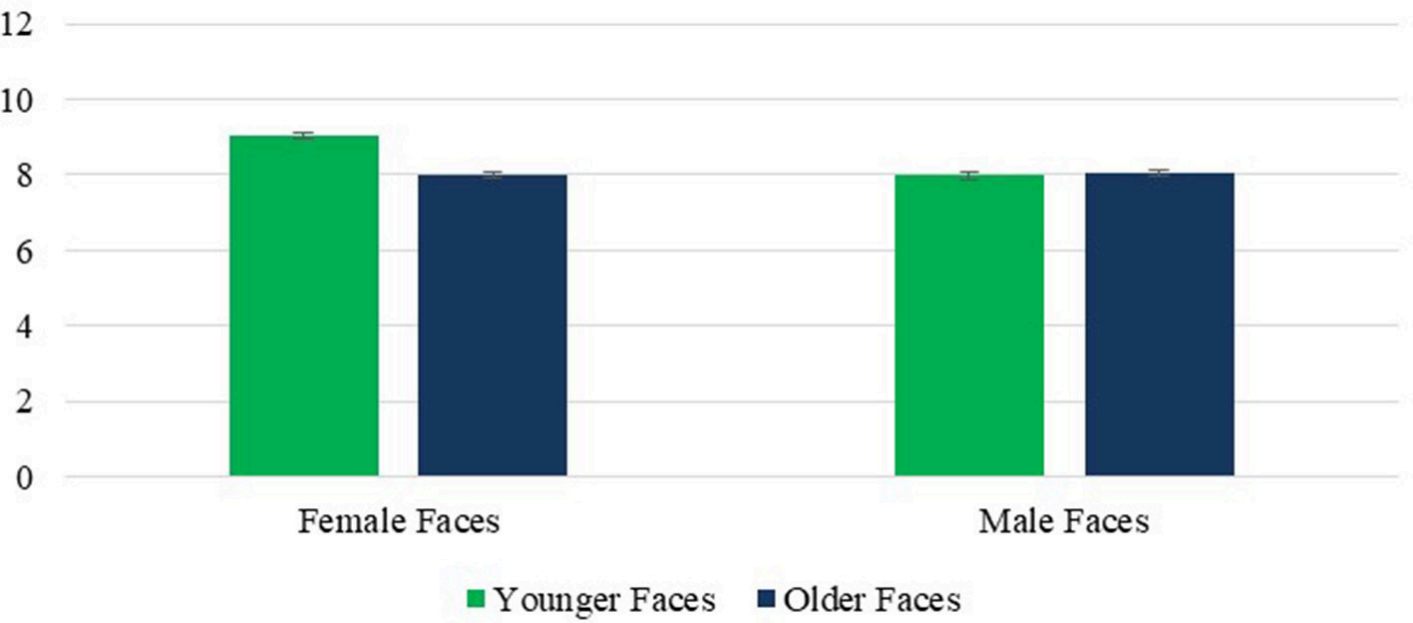
Warmth: Face Age × Face Sex interaction. Error bars are SE.

A main effect of face age revealed that, contrary to prediction, younger faces were rated as warmer than older faces, *F*_(1, 236)_ = 21.20, *p* < 0.001, η^2^ = 0.082. However, a significant face age × rater age interaction, *F*_(1, 236)_ = 125.77, *p* < 0.001, η^2^ = 0.348, revealed that this effect was moderated by rater age. It held true for older raters, *p* < 0.001, contrary to the expectation that OA might respond more positively to older faces, while younger raters attributed greater warmth to older faces than younger faces *p* = 0.044, consistent with previous research findings. The face age × rater age interaction further revealed a surprising other-age favoritism, with OA rating younger faces as warmer than YA did, with the reverse effect of rater age for older faces (Figure 2). Finally, a face age × face sex interaction, *F*_(1, 236)_ = 26.47, *p* < 0.001, η^2^ = 0.101, revealed that the perception of greater warmth in younger than older faces was significant for female faces, *p* < 0.001, but not for male faces, *p* = 0.703 (Figure 3).

A significant effect of face sex, *F*_(1, 236)_ = 24.01, *p* < 0.001, η^2^ = 0.092, reflected the perception of greater warmth in female than male faces, as predicted. However, this effect was moderated by the above noted interaction with face age, which revealed that the overall tendency to rate female faces as warmer than male faces was significant for younger faces, *p* < 0.001 but not older ones, *p* = 0.863. Finally, a significant main effect of rater age, *F*_(1, 236)_ = 4.41, *p* = 0.037, η^2^ = 0.018 revealed that OA had higher scores on the warmth composite than YA. The interaction effects of rater age × face sex and rater age × face sex × face age were not significant, respective *Fs*_(1, 236)_ = 0.75 and 0.87, *p*s = 0.386 and 0.353, η^2^ = 0.003 and 0.004.

##### Competence composite

**Figure 4:**
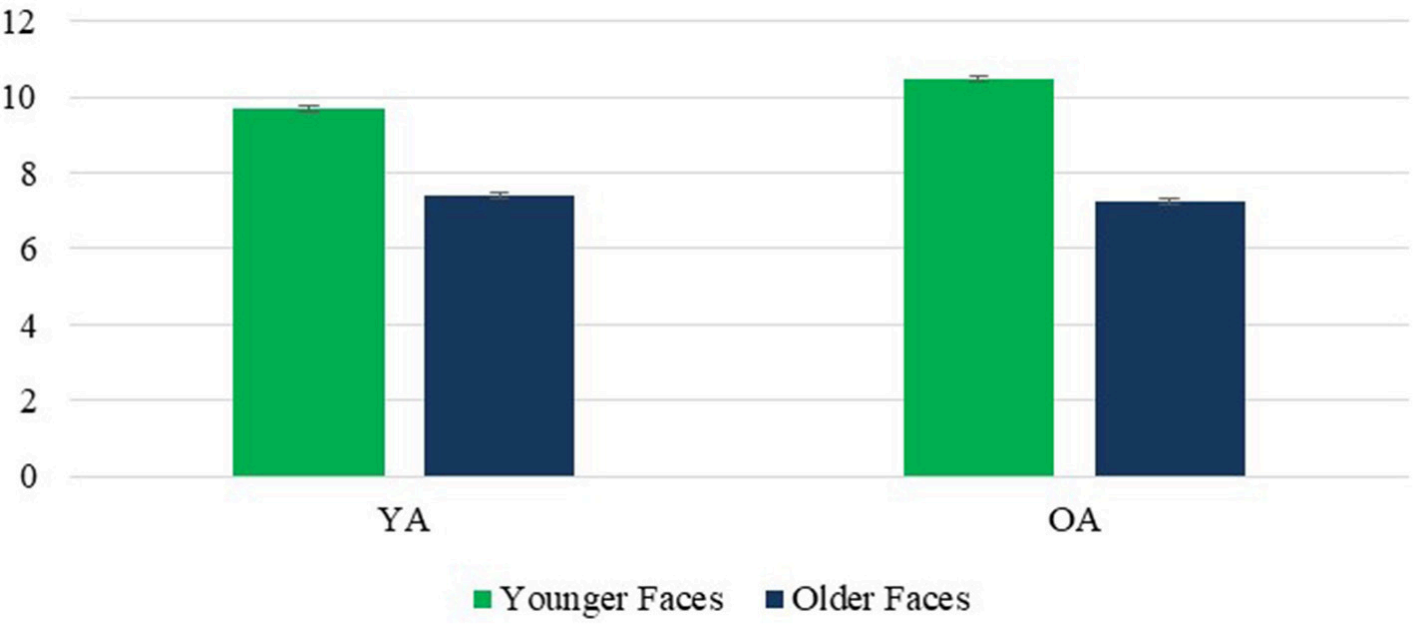
Competence: Face Age × Rater Age interaction. Error bars are SE.

**Figure 5:**
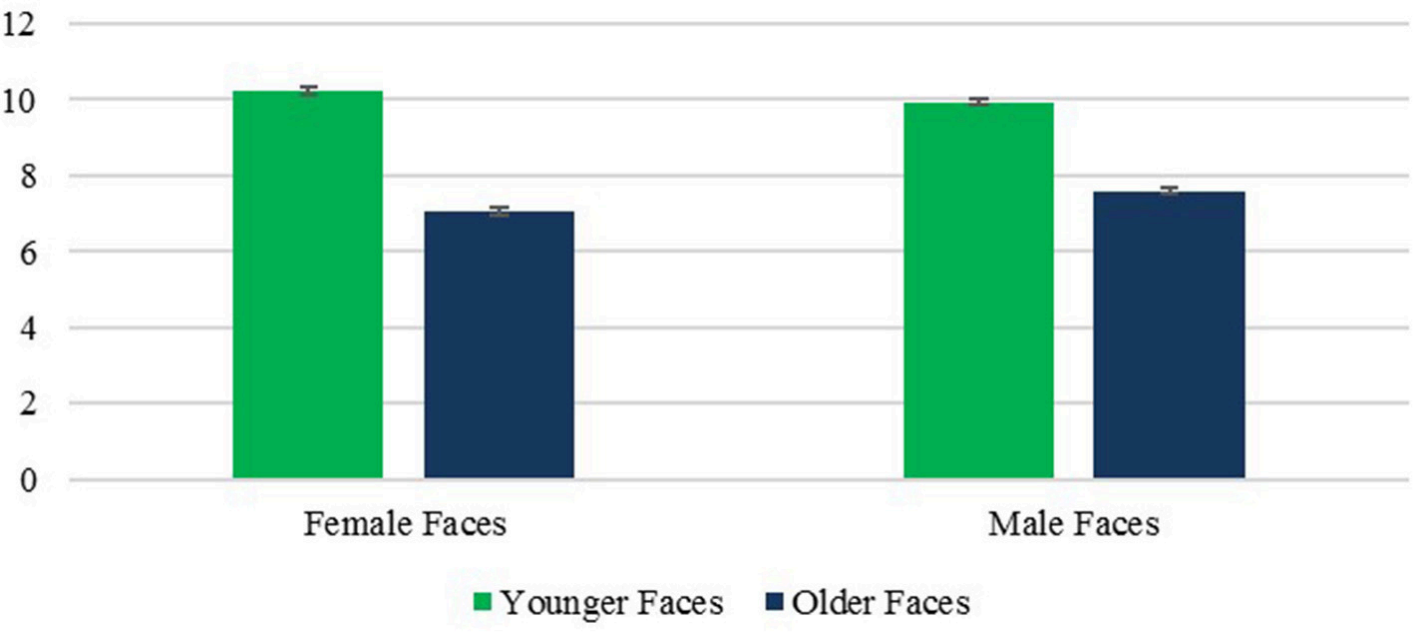
Competence: Face Age × Face Sex interaction. Error bars are SE.

A main effect of face age revealed that, as predicted, younger faces were rated as more competent than older faces *F*_(1, 236)_ = 622.77, *p* < 0.001, η^2^ = 0.725. A significant face age × rater age interaction, *F*_(1, 236)_ = 109.71, *p* < 0.001, η^2^ = 0.317 revealed that this effect was stronger for OA. However, the perception of greater competence in younger faces was highly significant for both groups, *ps* < 0.001 (Figure 4). What accounted for the interaction was an own-age favoritism, with YA rating younger faces as more competent than did OA, *p* < 0.001, and the reverse effect of rater age for older faces, *p* = 0.015.The perception of younger faces as more competent than older ones also held true for both male and female faces despite a significant face age × face sex interaction *F*_(1, 236)_ = 14.45, *p* < 0.001, η^2^ = 0.058, reflecting a stronger age effect for female faces, although the effects for both male and female faces were highly significant, both *ps* < 0.001 (Figure 5).

**Figure 6:**
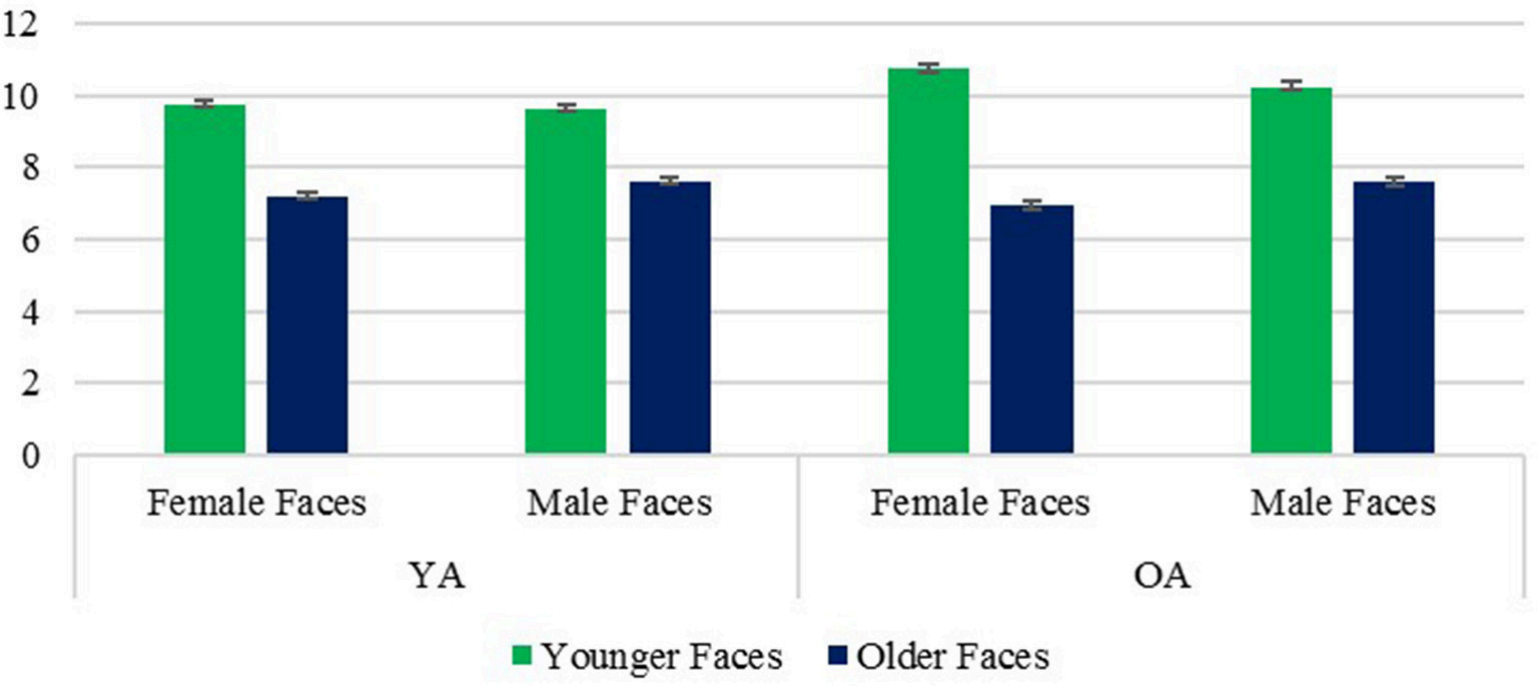
Competence: Face Age × Face Sex × Rater Age interaction. Error bars are SE.

Contrary to prediction, there was no significant effect of face sex on the competence composite scores, *F*_(1, 236)_ = 1.11, *p* = 0.292, η^2^ = 0.005. However, the face sex × face age interaction noted above revealed that younger women received higher scores than younger men *p* < 0.001, contrary to prediction, while older women received lower scores than older men, *p* = 0.003, as predicted. This interaction was qualified by a significant rater age × face age × face sex interaction, *F*_(1, 236)_ = 9.67, *p* < 0.001, η^2^ = 0.039, which revealed that the unexpected tendency for younger women to be rated as higher in competence than younger men held true for OA, *p* = 0.006., but not YA, *p* = 0.438, while the predicted perception of older women as less competent than older men held true for both OA and YA, respective *ps* < 0.001 and 0.010 (Figure 6). Finally, a significant effect of rater age, *F*_(1, 236)_ = 48.91, *p* < 0.001, η^2^ = 0.172, revealed that OA had higher scores on the competence composite than YA. The rater age × face sex interaction was not significant, *F*_(1, 236)_ = 0.56, *p* = 0.456, η^2^ = 0.002.

##### Attractiveness

**Figure 7:**
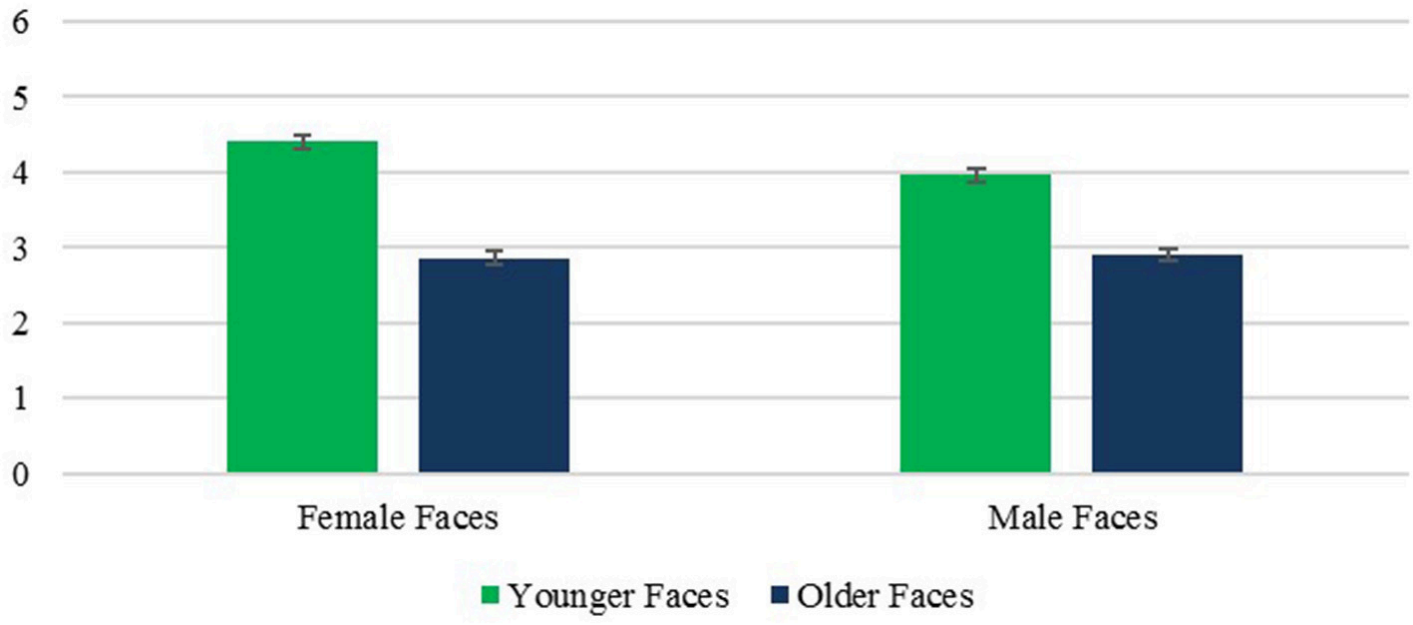
Attractiveness: Face Age × Face Sex interaction. Error bars are SE.

A main effect of face age revealed that, as predicted, older faces were rated as less attractive than younger faces, *F*_(1, 236)_ = 270.49, *p* < 0.001, η^2^ = 0.534. This effect did not vary with rater age *F*_(1, 236)_ = 1.66, *p* = 0.199, η^2^ = 0.007. It also held true for male faces, and female faces, both *ps* < 0.001, despite a significant face age × sex interaction *F*_(1, 236)_ = 10.04, *p* < 0.005, η^2^ = 0.041, which reflected a larger age difference for female faces (Figure 7).

An unexpected finding was that female faces were rated as more attractive than male faces, *F*_(1, 236)_ = 6.33, *p* < 0.05, η^2^ = 0.026, and the face age × sex interaction noted above revealed that this was true for younger faces, *p* < 0.001, but not older ones, *p* = 0.645. Finally, a significant effect of rater age, *F*_(1, 236)_ = 1,525.40, *p* < 0.001, η^2^ = 0.866, revealed that OA gave higher attractiveness ratings than YA. The rater age × face age effect was not significant, *F*_(1, 236)_ = 1.66, *p* = 0.199, η^2^ = 0.007, and neither was the rater age × face age effect, *F*_(1, 236)_ = 3.19, *p* = 0.075, η^2^ = 0.013, or the triple order interaction, *F*_(1, 236)_ = 0.15, *p* = 0.702, η^2^ = 0.001.

##### Summary

YA ratings were higher for older than younger faces on the warmth composite and lower for older than younger faces on the competence composite, consistent with the age stereotype “doddering but dear” documented in previous research. Like YA, OA ratings were lower for older than younger faces on the competence composite. However, they were also lower for older faces on the warmth composite, contrary to prediction. Whereas age stereotypes were moderated by rater age, sex stereotypes were moderated by face age. YA and OA ratings were higher for female than male faces on the warmth composite, as predicted, but this held true only for younger faces. In addition, whereas both YA and OA ratings were higher for male than female faces on the competence composite, this held true only for older faces. YA did not show this sex stereotype for younger faces and OA perceived younger women as more competent than younger men. As predicted, older faces were judged less attractive than younger ones. Unexpectedly, male faces were also judged less attractive than female faces, which means that we can examine the contribution of facial attractiveness to the gender stereotypes we've observed. Finally, OA gave more positive ratings than YA on the warmth and competence composites and attractiveness, consistent with other evidence for an OA positivity effect that includes trait impressions (Carstensen and Mikels, 2005; Zebrowitz et al., 2013; Mammarella et al., 2016, 2017). However, greater OA positivity was shown only for younger faces on the warmth composite and only for older faces on the competence composite.

#### Regressions predicting scores on the trait composites

We performed separate regression analyses on the warmth and competence composites for OA and YA to determine the contribution of emotion resemblance and attractiveness to their age and gender stereotypes. Step 1 of the regressions entered face age and face sex. Step 2 entered resemblance to happy, angry, and surprised faces. Step 3 entered face attractiveness. To facilitate readability, the regression results are presented in Table 5 (warmth composite) and Table 6 (competence composite) rather than in the text, where statistics are limited to comparisons of the change in the β*s* for face age and sex from one step to the next.

**Table 5:** Summary of regression analysis for variables predicting warmth.

	**Variable**	**Step 1**							**Step 2**							**Step 3**						
							**Correlation**							**Correlation**							**Correlation**	
		**B**	**SE B**	**β**	***t***	***p***	**Partial**	**Semi-partial**	**B**	**SE B**	**β**	***t***	***p***	**Partial**	**Semi-partial**	**B**	**SE B**	**β**	***t***	***p***	**Partial**	**Semi-partial**
YA	Face Age	0.254	0.13	0.124	1.97	0.050	0.127	0.124	0.234	0.17	0.114	1.37	0.172	0.890	0.085	0.635	0.19	0.309	3.31	0.001	0.212	0.198
	Face Sex	0.460	0.13	0.224	9.56	0.000	−0.225	−0.224	0.396	0.13	0.193	2.97	0.003	−0.190	−0.184	0.283	0.13	0.138	2.14	0.033	−0.139	−0.128
	Happy								−0.003	0.01	−0.038	−0.46	0.648	−0.030	−0.028	−0.002	0.01	−0.027	−0.34	0.733	−0.022	−0.020
	Angry								−0.004	0.01	−0.068	−0.95	0.341	−0.062	−0.059	−0.007	0.00	−0.114	−1.63	0.105	−0.106	−0.097
	Surprise								0.013	0.01	0.164	2.45	0.015	0.158	0.152	0.012	0.01	0.159	2.46	0.015	0.159	0.147
	Attractiveness															0.352	0.09	0.333	4.11	0.000	0.260	0.246
	*R*^2^	0.065							0.103							0.163						
	*R*^2^ Change	0.065							0.038							0.061						
	*F* for *R*^2^ Change	8.281							3.266							16.879						
	*df*	2,237							3,234							1,233						
	*p*	0.000							0.022							0.000						
OA	Face Age	−1.226	0.13	−0.509	−9.49	0.000	−0.525	−0.509	−1.201	0.17	−0.499	−6.91	0.000	−0.412	−0.372	0.356	0.16	0.148	2.22	0.028	0.144	0.085
	Face Sex	0.575	0.13	0.239	4.44	0.000	−0.277	−0.239	0.597	0.14	0.248	4.39	0.000	−0.276	−0.237	0.359	0.10	0.149	3.67	0.000	−0.234	−0.140
	Happy								0.002	0.01	0.017	0.24	0.811	0.016	0.013	−0.002	0.01	−0.027	−0.53	0.599	−0.034	−0.020
	Angry								0.006	0.01	0.078	1.26	0.208	0.082	0.068	−0.006	0.00	−0.079	−1.74	0.084	−0.113	−0.066
	Surprise								0.003	0.01	0.037	0.64	0.525	0.042	0.034	0.004	0.00	0.049	1.19	0.234	0.078	0.046
	Attractiveness															1.197	0.08	0.901	15.19	0.000	0.705	0.581
	*R^2^*	0.316							0.321							0.659						
	*R^2^*Change	0.316							0.005							0.338						
	*F* for *R*^2^ Change	54.859							0.561							230.646						
	*Df*	2,237							3,234							1,233						
	*p*	0.000							0.641							0.000						

*Higher scores for face age signify that older faces are rated higher and higher scores for face sex indicate that female faces are rated higher*.

**Table 6:** Summary of regression analysis for variables predicting competence.

	**Variable**	**Step 1**							**Step 2**							**Step 3**						
							**Correlation**							**Correlation**							**Correlation**	
		**B**	**SE B**	**β**	***t***	***p***	**Partial**	**Semi–partial**	**B**	**SE B**	**β**	***t***	***p***	**Partial**	**Semi–partial**	**B**	**SE B**	**β**	***T***	***p***	**Partial**	**Semi–partial**
YA	Face Age	−2.277	0.12	−0.783	−19.43	0.000	−0.784	−0.783	−2.225	0.16	−0.765	−14.17	0.000	−0.680	−0.571	−1.203	0.13	−0.413	−9.46	0.000	−0.527	−0.265
	Face Sex	−0.150	0.12	−0.052	−1.28	0.201	0.083	0.052	−0.102	0.12	−0.035	−0.83	0.407	0.054	0.033	−0.390	0.09	−0.134	−4.46	0.000	0.280	0.125
	Happy								0.001	0.01	0.007	0.12	0.902	0.008	0.005	0.003	0.00	0.025	0.68	0.499	0.044	0.019
	Angry								0.006	0.00	0.066	1.41	0.159	0.092	0.057	−0.001	0.00	−0.016	−0.50	0.616	−0.033	−0.014
	Surprise								−0.003	0.01	−0.025	−0.57	0.571	−0.037	−0.023	−0.004	0.00	−0.034	−1.11	0.267	−0.073	−0.031
	Attractiveness															0.895	0.06	0.599	15.79	0.000	0.719	0.443
	*R*^2^	0.615							0.621							0.817						
	*R*^2^ Change	0.615							0.005							0.196						
	*F* for change in *R*^2^	189.593							1.088							249.286						
	*df*	2,237							3,234							1,233						
	*p*	0.000							0.355							0.000						
OA	Face Age	−3.230	0.13	−0.854	−25.31	0.000	−0.854	−0.854	−3.161	0.17	−0.836	−18.63	0.000	−7.773	−0.624	−1.439	0.13	−0.380	−10.83	0.000	−0.579	−0.218
	Face Sex	−0.083	0.13	−0.022	−0.65	0.519	0.042	0.022	−0.010	0.13	−0.003	−0.08	0.940	0.005	0.003	−0.274	0.08	−0.072	−3.38	0.001	0.216	0.068
	Happy								0.001	0.01	0.010	0.21	0.831	0.014	0.007	−0.003	0.00	−0.021	−0.80	0.426	−0.052	−0.016
	Angry								0.008	0.01	0.067	1.74	0.083	0.113	0.058	−0.005	0.00	−0.043	−1.82	0.070	−0.118	−0.037
	Surprise								−0.006	0.01	−0.044	−1.23	0.221	−0.080	−0.041	−0.005	0.00	−0.036	−1.64	0.102	−0.107	−0.033
	Attractiveness															1.325	0.07	0.635	20.30	0.000	0.799	0.409
	*R*^2^	0.730							0.738							0.905						
	*R*^2^ Change	0.730							0.008							0.168						
	*F* for change in *R*^2^	320.505							2.281							412.202						
	*df*	2,237							3,234							1,233						
	*p*	0.000							0.080							0.000						

*Higher scores for face age signify that older faces are rated higher and higher scores for face sex indicate that female faces are rated higher*.

##### Warmth composite: YA

The tendency for YA ratings to be higher for older than younger faces on the warmth composite, lost significance when emotion resemblance indices were entered into the equation at Step 2, but this change in the age effect was not significant, *t* = 0.093 *p* = 0.92 (see Weaver and Wuensch, 2013). Higher YA ratings of female than male faces on the warmth composite, remained significant at Step 2, and the change in the sex effect was not significant, *t* = 0.35 *p* = 0.727. However, the *R*^2^ change at Step 2 was significant, reflecting a significant positive effect of surprise resemblance on perceived warmth. Although the effect of surprise resemblance was consistent with previous research findings, it did not influence the age or gender stereotypes shown on the warmth composite. Contrary to prediction, happy and anger resemblance had no significant effects on YA ratings on the warmth composite.

Adding attractiveness into the equation at Step 3 produced a significant *R*^2^ change. Not only was attractiveness a significant predictor of YA perceived warmth, but also including it in the model restored and strengthened the original effect of face age that had lost significance at Step 2, However, this increase in the β was not significant as compared with Step 2, *t* = 1.57, *p* = 0.116 or Step 1, *t* = 1.65, *p* = 0.098. Including attractiveness also had no significant influence on the effect of face sex, *t* = 0.44 *p* = 0.658. Resemblance to surprise continued to predict greater perceived warmth with attractiveness in the model, and the effects of resemblance to happy and angry expressions remained non-significant.

##### Warmth composite: OA

The tendency for OA to rate younger faces higher in warmth than older ones remained significant when emotion resemblance indices were entered into the equation at Step 2, and there was no significant change in the face age effect, *t* = 0.12, *p* = 0.907. Similarly, the tendency for OA to rate female faces higher in warmth than male faces remained significant at Step 2, and there was no significant change in the face sex effect, *t* = 0.11, *p* = 0.908. The *R*^2^ change also was not significant. Contrary to prediction, none of the emotion resemblance indicators predicted impressions of warmth.

Adding attractiveness into the equation at Step 3 produced a significant *R*^2^ change. Not only was attractiveness a significant predictor of perceived warmth, but also including it in the model reversed the perception of younger faces as warmer than older ones, yielding the pattern consistent with predictions, and this change in the β was significant, *t* = 3.70, *p* < 0.001. Although including attractiveness in the model also weakened the greater perceived warmth of female faces, as predicted, this change was not significant, *t* = 1.38 *p* = 0.168. Finally, there was a marginally significant negative effect of anger resemblance on perceived warmth at Step 3, consistent with predictions. However, there were no effects for resemblance to happy or surprise expressions.

##### Competence composite: YA

The significant tendency for YA to perceive higher competence in younger than older faces remained significant when the emotion resemblance indices were entered at Step 2, and the age effect did not change significantly, *t* = 0.05, *p* = 0.950. Perceptions of competence did not vary with face sex, and this remained true at Step 2. The *R*^2^ change at Step 2 also was not significant, and contrary to prediction, none of the emotion resemblance indicators predicted impressions of competence.

Adding attractiveness at Step 3 produced a significant *R*^2^ change., Not only did attractiveness have a significant positive effect on perceived competence, but also the negative effect of age on perceived competence was weaker with attractiveness in the model, as predicted, and this change in the β was significant, *t* = 4.96, *p* < 0.001. In addition, consistent with predictions, a significant effect emerged for face sex, showing higher perceived competence in male than female faces, and this change in the β was marginally significant, *t* = 1.92, *p* = 0.056. All of the emotion resemblance effects on perceived competence remained non-significant at Step 3.

##### Competence composite: OA

The significant tendency for OA to rate younger faces as more competent than older ones remained significant when the emotion resemblance indices were entered at Step 2, and the age effect did not change significantly, *t* = 0.32, *p* = 0.747. OA perceptions of competence did not vary with face sex, and this remained true at Step 2, The *R*^2^ change at Step 2 was not significant, and contrary to prediction, none of the emotion resemblance indicators predicted impressions of competence.

Adding attractiveness at Step 3 produced a significance *R*^2^ change. Not only did attractiveness have a significant positive effect on perceived competence, but also the positive effect of age on competence impressions was weakened with attractiveness in the model, as predicted, and this decrease in the β was significant, *t* = 8.05, *p* < 0.001. In addition, with the higher attractiveness of female than male faces controlled, a significant effect emerged for face sex, with higher perceived competence in male than female faces, and this change in the β was marginally significant, *t* = 1.73, *p* = 0.085. The emotion resemblance effects on perceived competence remained non-significant at step 3.

##### Summary

Controlling for emotion resemblance did not influence the effects of face age or face sex on perceived warmth and competence. However, surprise resemblance did increase YA impressions of warmth across all faces, and anger resemblance marginally decreased OA warmth impressions, while none of the emotion resemblance indices affected impressions of competence^2^. In addition, in contrast to the null effects of emotion resemblance on age and gender stereotypes, controlling attractiveness, which was greater in younger than older and female than male faces, had a significant influence. In the case of age stereotypes, controlling attractiveness replaced OA perception of greater warmth in younger than older faces, with the perception of greater warmth in older faces, a significant reversal. Controlling attractiveness also significantly decreased YA and OA impressions of greater competence in the younger faces. In the case of gender stereotypes, it was only when controlling the greater attractiveness of female than male faces, that YA and OA showed greater perceived competence in male than female faces, changes that were marginally significant.

### Discussion

#### Age and gender stereotypes

Previous research showing less favorable evaluations of older than younger individuals on a competence dimension, with the reverse on a warmth dimension (Cuddy et al., 2008) were confirmed by the trait impressions of YA in our study. Whereas YA thus perceived older faces as “doddering but dear,” OA judged younger faces more favorably on the warmth composite, rather than older faces. As discussed below, this reversal of the effect predicted from previous research was driven by the greater attractiveness of the younger faces, which was not a salient cue in research examining age stereotypes from category labels (Cuddy and Fiske, 2002; Fiske et al., 2002; Cuddy et al., 2008). Our results suggest that the YA stereotype of older faces as warmer is robust in the face of their lesser attractiveness, whereas the OA stereotype is not.

The trait impressions of both YA and OA confirmed previous research showing gender stereotypes paralleling age stereotypes, with less favorable evaluations of women than men on a competence dimension and the reverse on a warmth dimension (Fiske et al., 2002; Cuddy et al., 2008). However, these effects were moderated by face age, with higher female warmth scores shown only for younger faces and lower female competence scores shown only for older faces. As discussed below, the greater attractiveness of younger female than male faces contributed to these effects.

We did not select male and female faces with the intention of creating variation in attractiveness. Whether the gender differences we found for younger but not older faces in our sample generalize to a more representative sample is worthy of further investigation. If they are representative, this would have implications for understanding changes in gender stereotypes across age. It should be noted that only a handful of previous studies have examined age and gender stereotypes as a function of the person's position on the other dimension, and to our knowledge none have done so using impressions of faces (for a review, see Andreoletti et al., 2015). Our results urge caution in generalizing from YA age and gender stereotypes of younger faces to older faces or older raters.

#### Contribution of emotion resemblance to age and gender stereotypes

Face age and sex differences in emotion resemblance had no effect on age or gender stereotypes as evidenced by no changes in the effects of age and gender on perceived warmth and competence when controlling emotion resemblance. One possible explanation for our null results is that differences in the emotion resemblance of older vs. younger adults or women vs. men that were detected by the connectionist models were not strong enough to override the influence of other facial information provided in the photographs. Another possible explanation is that the emotion resemblance differences were not strong enough to override the influence of cultural stereotypes unrelated to appearance. Notably, however, previous research found that structural resemblance to emotions did moderate race stereotypes (Zebrowitz et al., 2010). The divergent results may reflect a stronger influence of variations in attractiveness among faces in the present study than those varying in race.

Although emotion resemblance did not contribute to age or gender stereotypes, we did find effects of emotion resemblance on trait impressions across all faces. Specifically, surprise resemblance increased YA ratings of faces on the warmth composite, and anger resemblance marginally decreased OA ratings, thus establishing the validity of these predictors. These effects of emotion resemblance extend previous research that included only younger faces, although YA and OA perceivers showed both effects in one study (Zebrowitz et al., 2007, 2010; Franklin and Zebrowitz, 2013).

#### Contribution of attractiveness to age and gender stereotypes

Age differences in attractiveness contributed to age stereotypes on both the warmth and competence dimensions. The unexpected tendency for younger faces to be judged warmer by OA was significantly reversed with attractiveness controlled, indicating that statistically equating the attractiveness of younger and older faces uncovered this OA positive stereotype of older individuals which was shown by YA without controlling attractiveness. In the case of competence stereotypes, the higher scores for younger faces by both YA and OA became significantly weaker with attractiveness controlled, indicating that the higher attractiveness of younger than older faces contributed to this negative stereotype of older individuals.

Gender differences in attractiveness in our study also contributed to gender stereotypes. In the case of competence stereotypes, there were no significant effects of face sex until attractiveness was controlled, yielding significantly higher perceived competence of male faces by both YA and OA, changes that were marginally significant. This indicates that the greater attractiveness of women than men in the present study masked a tendency to perceive men as more competent. Similarly, the tendency for both YA and OA to judge female faces as warmer was weakened with the greater attractiveness of women statistically controlled, although changes in these effects were not significant.

The effects of attractiveness on age stereotypes is consistent with previous research that found that older faces' greater resemblance to unattractive, anomalous faces partly explained the tendency to rate them as less sociable, warm and healthy than younger faces (Zebrowitz et al., 2003). The present results also demonstrate that this evidence for a contribution of age differences in attractiveness to YA negative age stereotypes generalizes to OA judges and to a much larger sample of faces. Furthermore, the present findings show that these effects of age differences in attractiveness on age stereotypes are independent of differences in the emotion resemblance of younger and older faces, which were controlled in the regression models.

Although it is unclear whether the greater attractiveness of younger female than male faces in our study has any generalizability to other samples, it is likely that most samples of older faces will be perceived as less attractive than younger ones. As such, our results have important implications for addressing age biases. In particular, our finding that age differences in attractiveness make a substantial contribution to negative stereotypes of older people's competence is consistent with evidence that age-appearance has a stronger effect on simulated personnel decisions than does chronological age (Kaufmann et al., 2016). This appearance bias is particularly troubling given evidence that attractiveness is not a reliable cue to the competence of older people. Although it was related to self-reported physical fitness in older people, it was unrelated to their reasoning or short term memory, albeit positively related for younger people (Zebrowitz et al., 2014). These results suggest that policies to combat age discrimination in the workplace should prioritize selection processes that keep personnel officers blind to applicants' appearance as long as possible. Although this may seem far-fetched, the fact is that major symphony orchestras have implemented audition procedures in which the applicant performs behind a screen so that the judges are ignorant of demographic characteristics (Goldin and Rouse, 2000). Our finding that age differences in attractiveness also have effects on the perception of greater warmth in older than younger adults may also have important practical implications

## Summary and implications

Our results document age differences in the resemblance of neutral expression faces to emotion expressions and extend previous evidence for gender differences to include older faces. However, emotion resemblance did not contribute to age or gender stereotypes, although it did influence impressions of warmth across all faces. Our results also extend previous evidence that YA perceive older adults and women as warmer and less competent than younger adults and men, respectively. Specifically, we found variations in age stereotypes across perceiver age and variations in gender stereotypes across face age. These moderating effects provide a caveat to conclusions from the large body of stereotype research that generalizes from YA impressions and ignores cross-cutting demographic categories. Our results also provide a caveat to conclusions about age and gender stereotypes derived from responses to category labels. Our assessment of stereotypes from trait impressions of faces as opposed to category labels revealed significant impacts of age and gender differences in attractiveness, as evidenced by changes in the stereotypes with attractiveness controlled. Specifically, the lower attractiveness of older faces weakened OA perceptions of their greater warmth as compared with younger faces and strengthened both YA and OA perceptions of their lesser competence. Similarly, the greater attractiveness of female faces in our study weakened their lesser perceived competence as compared with male faces. These results reveal the importance of assessing stereotypes with a methodology that is sensitive to influences of group differences in appearance that can exacerbate or mitigate stereotypes in more ecologically valid contexts. Positive stereotypes of older adults' warmth may have little effect in contexts where their lower attractiveness is salient, whereas negative stereotypes of older adults' competence may be exacerbated in such contexts.

## Ethics statement

This study was carried out in accordance with the recommendations of the ethical committee of the University of Chieti with written informed consent from all subjects. All subjects gave written informed consent in accordance with the Declaration of Helsinki. The protocol was approved by the University of Chieti committee.

## Author contributions

Data collection: RP; Data analyses: RP, LZ; Conceptualization: LZ, RA, RK, UH, RP; Writing RP, LZ.

### Conflict of interest statement

The authors declare that the research was conducted in the absence of any commercial or financial relationships that could be construed as a potential conflict of interest.

## Footnotes

^1^Although the mean ratings of the faces confirmed that they had neutral expressions with no sex or age differences, we also examined activation for a subset of 191 faces that had mean smile ratings no greater than 1.50. The results were identical to the full set.

^2^In addition to the effects of surprise on warmth impressions across all faces with face age and sex controlled, we found several significant or marginally effects of emotion resemblance within the age/sex categories, which we summarize below for the interested reader. The direction of the effects was often variable across levels of face age and sex as well as across rater age. One notable difference was that anger resemblance enhanced perceived competence for young faces but reduced it for old faces. *Young female faces*: Anger resemblance showed a positive relationship to OA competence ratings, *r*_(60)_ = 0.282, *p* = 0.029 and surprise resemblance showed a marginal negative relationship, *r*_(60)_ = −0.250, *p* = 0.054. *Young male faces*: anger resemblance showed a positive relationship to OA and YA competence ratings, respective *rs*_(60)_ = 0.378 and 0.419, *p*s = 0.003 and 0.001, anger resemblance also showed a positive relationship to OA warmth ratings, *r*_(60)_ = 0.300, *p* = 0.020, while surprise resemblance showed a positive relationship to YA warmth ratings, *r*_(60)_ = 0.333, *p* = 0.009. *Old female faces:* Anger resemblance showed a negative relationship to YA warmth ratings, *r*_(60)_ = −0.300, *p* = 0.020, and surprise resemblance showed a positive relationship, *r*_(60)_ = 0.446, *p* < 0.001. Surprise resemblance also showed a marginal negative relationship to OA competence ratings, *r*_(60)_ = −0.228, *p* = 0.080. *Old male faces:* Anger resemblance showed a negative relationship to competence ratings, that was significant for YA, *r*_(60)_ = −0.290, *p* = 0.025, and marginal for OA, *r*_(60)_ = −0.220, *p* = 0.091. Although these findings are beyond the scope of the hypotheses we tested, the warrant further investigation.
